# BATF2-mediated control of astrocyte proliferation

**DOI:** 10.1016/j.jbc.2025.110710

**Published:** 2025-09-12

**Authors:** Rachel A. Tinkey, Benjamin J. Frostino, Maria L. Habean, Jessica L. Williams

**Affiliations:** 1 Department of Neurosciences, Cleveland Clinic, Cleveland, Ohio, USA; 2 School of Biomedical Sciences, Kent State University, Kent, Ohio, USA; 3 College of Science, University of Notre Dame, South Bend, Indiana, USA; 4 Department of Neurosciences, Case Western Reserve University, Cleveland, Ohio, USA

**Keywords:** BATF2, astrocyte, cell proliferation, transcription factor, chromatin immunoprecipitation, cyclin D1, CDK

## Abstract

Astrocyte proliferation in the central nervous system is tightly controlled and is driven by the coordinated expression of regulatory proteins, including cyclins and cyclin-dependent kinases (CDKs), that dictate cell cycle progression. While most of the postnatal proliferation in the central nervous system occurs in well-defined stem cell niches, proliferation of differentiated glial cells can also be observed to maintain local populations during homeostasis and in response to inflammation. However, the transcriptional programs that regulate homeostatic proliferation of terminally differentiated astrocytes are not fully understood. Here, we identify a novel basic leucine zipper ATF-like transcription factor 2 (BATF2) as a prominent regulator of cell cycle genes in astrocytes. Specifically, loss of BATF2 resulted in increased expression of proliferation proteins, including Ki67 and phospho-histone H3. Further, chromatin immunoprecipitation sequencing revealed that BATF2 binds to regulatory regions of several cell cycle–related genes that encode CDK regulatory subunit 1B, CDK2, and cyclin D1 . Concomitantly, we found that deletion of BATF2 increased transcription of these target genes. In addition, we examined the relationship of BATF2 and cyclin D1 in patient-derived glioblastoma samples and found that elevated levels of BATF2 had a corresponding decrease in cyclin D1. Collectively, our study demonstrates that BATF2 participates in the control of astrocytic cell cycle gene expression and further highlights BATF2 as a suppressor of uncontrolled proliferation.

Astrocyte proliferation is a hallmark response to central nervous system (CNS) injury and inflammation in a variety of contexts. Upon CNS insult, astrocytes can rapidly proliferate, contributing to glial scar formation that aids in the sequestration of damaged tissue and initiation of tissue repair (1, 2, 3, 4, 5, 6, 7, 8). While a response to inflammatory stimuli is a primary driver of astrocyte proliferation in the adult CNS, astrocytes maintain the ability to respond to mitogenic signals under physiological conditions (9, 10, 11). Notably, mature hippocampal astrocytes retain their proliferative ability, generating new astrocytes through local cell division (12). This pattern of proliferation by differentiated astrocytes has been demonstrated in other CNS regions as well as the cortex is a key source of new, postnatal glia resulting from the symmetric division of mature astrocytes (10). Similarly, adult astrocytic proliferation occurs in the spinal cord white matter, where astrocytes divide to maintain local populations (13). However, the mechanisms regulating the proliferative capacity of terminally differentiated astrocytes are not well understood.

At the molecular level, cell division and proliferation are modulated by a subgroup of cyclin-dependent kinases (CDKs), including CDK1, CDK2, CDK3, CDK4, and CDK6, that regulate cell cycle progression (14, 15). During somatic cell division, CDKs are often constitutively expressed in inactive forms and are activated through a series of phosphorylation events and interactions with cyclins (16, 17). Briefly, CDK4 and CDK6 are expressed in the G1 phase of the cell cycle, in which the cell receives mitogenic signals to begin division. Activation of these CDKs depends on interaction with D-type cyclins, which encompass cyclin D1, 2, and 3 (18, 19). Importantly, cyclin D2 and D3 are expressed highest in hematopoietic cell lineages, whereas cyclin D1 is expressed within embryonic tissue and maintains long-term expression in various adult tissues, including the brain and spinal cord (17, 20). Transition from G1 to S phase, in which DNA is replicated, and subsequent progression into G2 phase is primarily regulated by CDK2 and its interaction with cyclin E (21, 22, 23). Completion of the cell cycle and eventual division *via* the G2 and M phases is then regulated by CDK1 interactions with both A- and B-type cyclins (24, 25, 26, 27).

While the complexing of CDKs and cyclins is necessary for entry into and completion of the cell cycle, several other proteins aid in their regulation, including CDK inhibitors such as INK4 and the CDK-interacting protein/kinase inhibitory protein families. These proteins work to suppress CDK activation through blockage of catalytic domains (28, 29, 30, 31). Additional regulators of the cell cycle include cyclin-dependent kinase regulatory subunit (CKS) proteins, such as CKS1B, which bind to the catalytic subunits of CDKs and increase proliferation when overexpressed (32, 33, 34). Given the complexity of cell cycle processes, such as CDK regulation, providing insight into novel regulators of individual cell cycle components is crucial for understanding the mitotic process.

Recently, we identified basic leucine zipper ATF-like transcription factor (BATF)2 as a key modulator of neuroinflammatory responses in astrocytes (35); however, it is also thought of as necessary for maintenance of nonpathological cellular proliferation. Specifically, BATF2 dimerization with activator protein 1 (AP-1) subunits has been shown to suppress cell proliferation across several cancer types, and reduced expression or abnormal cellular localization of BATF2 contributed to increased tumor growth and the expression of cell cycle progression genes (15, 36, 37, 38). Further, BATF2 also decreased myeloid-derived suppressor cell recruitment, which contributed to reduced glioma growth, suggesting additional roles in modulating pathological microenvironments within the CNS (39). Given that the downregulation of BATF2 contributes to increased proliferation in several contexts, we sought to understand if BATF2 may play a role in regulating the homeostatic cell division of astrocytes.

In this study, we demonstrate that astrocytes deficient in BATF2 express increased proliferation proteins, including Ki67 and phospho-histone H3, compared with wildtype controls at baseline. Chromatin immunoprecipitation (ChIP) sequencing of human astrocytes revealed that BATF2 binds to motifs associated with genes responsible for positive regulation of the cell cycle and mitotic progression. These genes include *CCND1*, *CDK2*, and *CKS1B*, which are upregulated in *Batf2*^−/−^ astrocytes under basal conditions. Further, we demonstrate that cells overexpressing BATF2 have reduced expression of BATF2-regulated cell cycle genes and that siRNA knockdown of *BATF2* leads to subsequent upregulation of these markers. We also evaluated BATF2 and cyclin D1 expression within glioblastoma multiforme (GBM) patient samples to assess if a similar relationship may exist in disease. Analysis of publicly available data from The Cancer Genome Atlas in GBM revealed that patients with high *BATF2* expression had improved survival outcomes compared with those with low expression, and that overall *BATF2* transcript levels were elevated in GBM samples relative to nontumor controls. In addition, we demonstrated a negative correlation in which increased BATF2 levels led to decreased cyclin D1 at both the transcript and protein levels in GBM. Taken together, these data suggest that BATF2 functions as a homeostatic regulator of mitotic gene expression and aids in the maintenance of physiological astrocytic cell division, which can become aberrant during CNS pathology.

## Results

### BATF2 moderates astrocyte proliferation

**Figure 1:**
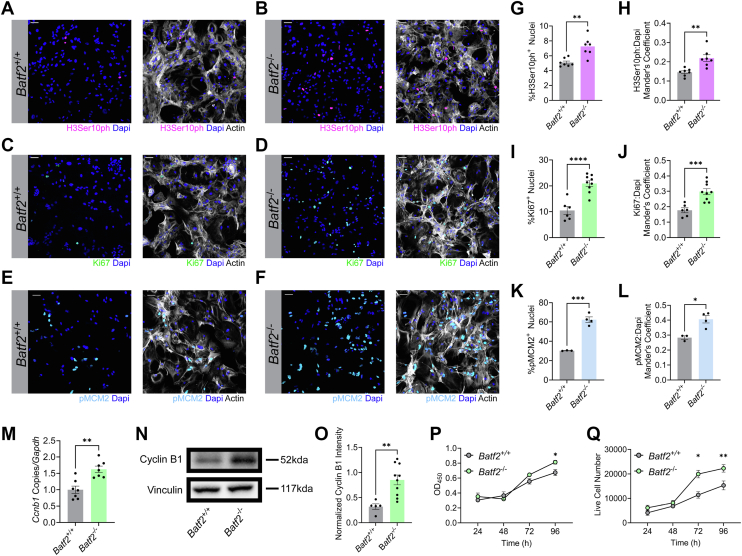
**Loss of BATF2 increases expression of proliferative markers in astrocytes.***A* and *B,* wildtype (*A*) and *Batf2*^−/−^ (*B*) astrocytes labeled for H3Ser10ph, actin, and nuclei counterstained with DAPI. The scale bars represent 50 μm. *C* and *D,* wildtype (*C*) and *Batf2*^−/−^ (*D*) astrocytes labeled for Ki67, actin, and nuclei counterstained with DAPI. The scale bars represent 50 μm. *E* and *F,* wildtype (*E*) and *Batf2*^−/−^ (*F*) astrocytes labeled for pMCM2, actin, and nuclei counterstained with DAPI. The scale bars represent 50 μm. *G,* quantification of H3Ser10ph-positive nuclei for wildtype and *Batf2*^−/−^ mice. Data were normalized to the total nuclei count, and data points are representative of individual mice. ∗∗*p* < 0.01 compared with wildtype samples by two-tailed Student's *t* test. The bars represent mean ± SEM. *H,* quantification of colocalization between H3Ser10ph and DAPI. Data points are representative of individual mice. ∗∗*p* < 0.01 compared with wildtype samples by two-tailed Student's *t* test. The bars represent mean ± SEM. *I,* quantification of Ki67-positive nuclei for wildtype and *Batf2*^−/−^ mice. Data points were normalized to the total nuclei count and are representative of individual mice. ∗∗∗∗*p* < 0.0001 compared with wildtype samples by two-tailed Student's *t* test. The bars represent mean ± SEM. *J,* quantification of colocalization between Ki67 and DAPI. Data points are representative of individual mice. ∗∗∗*p* < 0.001 compared with wildtype samples by two-tailed Student's *t* test. The bars represent mean ± SEM. *K,* quantification of pMCM2-positive nuclei for wildtype and *Batf2*^−/−^ mice. Data points were normalized to the total nuclei count and are representative of individual mice. ∗∗∗*p* < 0.001 compared with wildtype samples by two-tailed Student's *t* test. The bars represent mean ± SEM. *L,* quantification of colocalization between pMCM2 and DAPI. Data points are representative of individual mice. ∗*p* < 0.05 compared with wildtype samples by two-tailed Student's *t* test. The bars represent mean ± SEM. *M,* quantification of *Ccnb1* transcript expression in wildtype and *Batf2*^−/−^ astrocytes. Data points were normalized to the wildtype average and are representative of individual mice. ∗∗*p* < 0.01 compared with wildtype samples by two-tailed Student's *t* test. The bars represent mean ± SEM. *N,* representative Western blot of whole-cell cyclin B1 and vinculin protein levels of wildtype and *Batf2*^−/−^ astrocytes. *O,* quantification of whole-cell cyclin B1 protein levels in wildtype and *Batf2*^−/−^ astrocytes shown in *N*, normalized to vinculin expression. Data points are representative of individual mice. ∗∗*p* < 0.01 compared with wildtype samples by two-tailed Student's *t* test. The bars represent mean ± SEM. *P,* cell viability assay of wildtype and *Batf2*^−/−^ astrocytes over 96 h. Data points are representative of the combined average of individual mouse absorbance values at 450 nm (*Batf2*^+/+^*N* = 4, *Batf2*^−/−^*N* = 4 per timepoint). ∗*p* < 0.05 compared with wildtype samples by one-way ANOVA. The bars represent mean ± SEM. Q, live cell counts of wildtype and *Batf2*^−/−^ astrocytes over 96 h. Data points are representative of the combined average of individual mouse live cell counts (*Batf2*^+/+^*N* = 4, *Batf2*^−/−^*N* = 4 per timepoint). ∗*p* < 0.05, ∗∗*p* < 0.01 compared with wildtype samples by one-way ANOVA. The bars represent mean ± SEM. BATF2, basic leucine zipper ATF-like transcription factor 2; DAPI, 4′,6-diamidino-2-phenylindole; pMCM2, phospho-mini chromosome maintenance protein 2.

To determine if BATF2 may play a role in regulating cell division and/or proliferation in astrocytes, we initially assessed if loss of BATF2 resulted in altered mitosis and proliferation *via* labeling for phospho-histone H3, Ki67, and phospho-mini chromosome maintenance protein (pMCM)2, respectively. Primary wildtype and *Batf2*-deficient murine astrocytes were isolated and cultured *in vitro* and allowed to reach 70 to 80% confluency. Cells were then fixed and probed for phospho-histone H3 (Ser10) (H3Ser10ph), Ki67, and pMCM2 *via* immunocytochemistry (Fig. 1, *A*–*F*). *Batf2*^−/−^ astrocytes expressed increased nuclear levels of both phospho-histone H3 (Fig. 1, *G* and *H*), Ki67 (Fig. 1, *I* and *J*), and pMCM2 (Fig. 1, *K* and *L*) compared with wildtype controls. Transcript and protein levels of cyclin B1, a key regulator of the G2 and M phases of the cell cycle, were also found to be upregulated in *Batf2*^−/−^ astrocytes compared with controls, suggesting an increase in mitotic activity (Fig. 1, *M*–*O*). To further demonstrate that BATF2 loss promotes increased proliferation, the growth of *Batf2*^+/+^ and *Batf2*^−/−^ astrocytes was evaluated over 96 h using a cell viability assay and *via* live cell counts using trypan blue exclusion. Notably, Batf2^−/−^ cells exhibited increased viability and a higher number of live cells compared with wildtype controls (Fig. 1, *P* and *Q*), indicating a heightened proliferative state.

### BATF2 directly binds to cell cycle motifs in astrocytes

Some studies have linked BATF2 to the suppression of cell cycle regulation genes, including *CCND1*, through binding with AP-1 subunits in the context of cancer (15, 37). However, it is unknown if BATF2 functions similarly in astrocytes or if BATF2 may also confer regulation *via* direct DNA binding in addition to known protein-to-protein interactions. To assess this, we were initially interested in understanding if BATF2 was expressed by astrocytes that were undergoing active cell division. We found that BATF2 was coexpressed with phospho-histone H3 in human astrocytes, and more astrocyte nuclei were double positive for both markers compared with BATF2 alone (Fig. S1, *A*–*C*). Interestingly, the total number of phospho-histone H3 single-positive cells remained high; however, BATF2 expression correlated with increased phospho-histone H3 intensity (Fig. S1, *D*–*G*), which is known to rise during mitotic progression and is associated with chromosome condensation (40, 41). These data suggest that under basal conditions, BATF2 may be preferentially expressed in proliferating cells and that its expression could also be associated with specific stages of mitosis.

**Figure 2:**
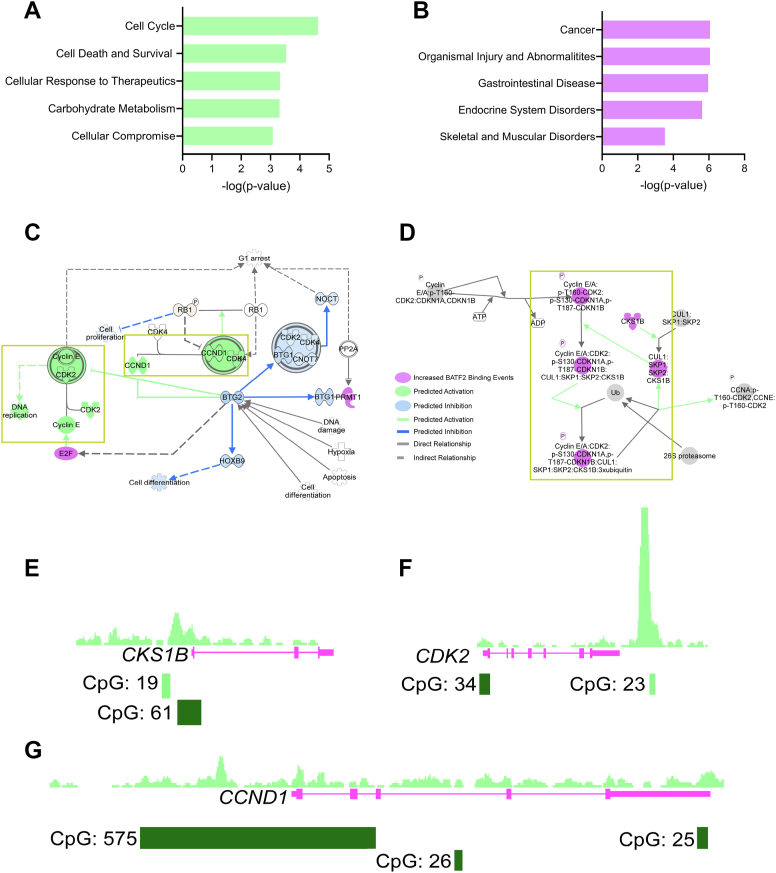
**BATF2 binds to DNA motifs associated with cell cycle genes in astrocytes.***A* and *B,* IPA of top-regulated molecular functions (*A*) and diseases (*B*) by BATF2 in human astrocytes. *C,* adapted IPA S phase pathway. *D,* adapted IPA cell cycle regulation by BTG protein pathway. *E–G,* ChIP sequencing peak region counts of BATF2-binding events at the *CKS1B* (*E*)*, CDK2* (*F*)*,* and *CCND1* (*G*) gene loci and CpG islands in human astrocytes. BATF2, basic leucine zipper ATF-like transcription factor 2; ChIP, chromatin immunoprecipitation; IPA, ingenuity pathway analyses.

To further determine if BATF2 binds to DNA motifs associated with cell cycle regulation, we utilized our recently reported ChIP-Seq dataset, where we found that in media-treated astrocytes, BATF2 bound to motifs associated with cellular processes such as the cell cycle and initiation of transcription (35). In this study, we expanded upon these findings and performed additional ingenuity pathway analyses (IPA) on annotated genes bound by BATF2 to identify specific pathways that were regulated by BATF2 at baseline. IPA identified cancer among the top associated diseases and the cell cycle as the most highly regulated molecular function of genes bound by BATF2 (Fig. 2, *A* and *B*). In-depth analysis of several pathways associated with the cell cycle (Fig. 2, *C* and *D*) identified *CKS1B*, *CDK2*, and *CCND1* as common genes bound by BATF2 (Fig. 2, *E*–*G*). These binding sites were also rich in CpG island regulatory sites, suggesting that cell cycle genes are, at least in part, directly regulated by BATF2 at the transcriptional level in astrocytes.

### BATF2 DNA binding results in a downregulation of cell cycle proteins

**Figure 3:**
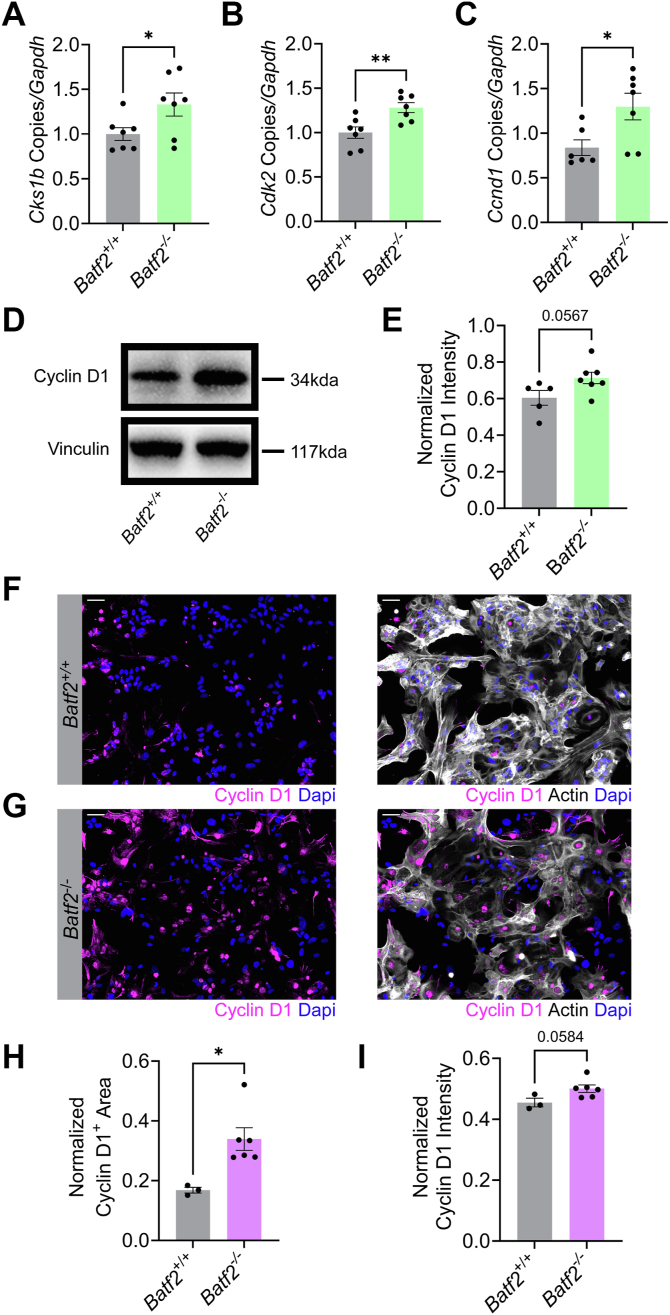
**BATF2 regulates cell cycle machinery expression in astrocytes.***A–C,* quantification of *Cks1b* (*A*), *Cdk2* (*B*), and *Ccnd1* (*C*) transcript expression in wildtype and *Batf2*^−/−^ astrocytes. Data points were normalized to the wildtype average and are representative of individual mice. ∗*p* < 0.05, ∗∗*p* < 0.01 compared with wildtype samples by two-tailed Student's *t* test. The bars represent mean ± SEM. *D,* representative Western blot of whole-cell cyclin D1 and vinculin protein levels in wildtype and *Batf2*^−/−^ astrocytes. *E,* quantification of whole-cell cyclin D1 protein levels in wildtype and *Batf2*^−/−^ astrocytes shown in *D*, normalized to vinculin expression. Data points are representative of individual mice. The bars represent mean ± SEM. *F* and *G,* wildtype (*F*) and *Batf2*^−/−^ (*G*) astrocytes labeled for cyclin D1, actin, and nuclei counterstained with DAPI. The scale bars represent 50 μm. *H* and *I,* quantification of cyclin D1-positive area (*H*) and intensity (*I*) for wildtype and *Batf2*^−/−^ mice. Data points were normalized to actin expression and are representative of individual mice. ∗*p* < 0.05 compared with wildtype samples by two-tailed Student's *t* test. The bars represent mean ± SEM. BATF2, basic leucine zipper ATF-like transcription factor 2; DAPI, 4′,6-diamidino-2-phenylindole.

**Figure 4:**
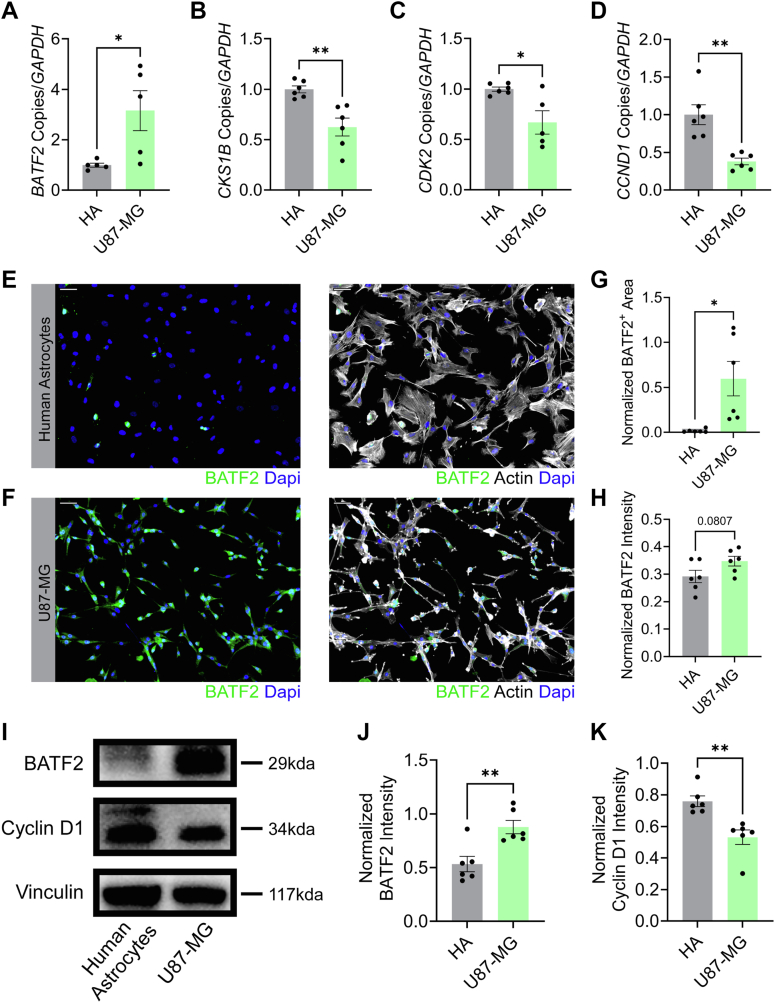
**Overexpression of BATF2 in U87-MG cells limits the expression of target cell cycle genes.***A–D,* quantification of *BATF2* (*A*), *CKS1B* (*B*), *CDK2* (*C*), and *CCND1* (*D*) gene expression in human astrocytes and U87-MG cells. Data points were normalized to the human astrocyte average and are representative of replicates from two independent experiments. ∗*p* < 0.05, ∗∗*p* < 0.01 compared with human astrocyte samples by two-tailed Student's *t* test. The bars represent mean ± SEM. *E* and *F,* human astrocytes (*E*) and U87-MG cells (*F*) labeled for BATF2, actin, and nuclei counterstained with DAPI. The scale bars represent 50 μm. *G* and *H,* quantification of BATF2-positive area (*G*) and intensity (*H*) for human astrocytes and U87-MG cells. Data points were normalized to actin expression and are representative of replicates from two independent experiments. ∗*p* < 0.05 compared with human astrocyte samples by two-tailed Student's *t* test. The bars represent mean ± SEM. *I,* representative Western blot of whole-cell BATF2, cyclin D1, and vinculin protein levels in human astrocytes and U87-MG cells. *J–K,* quantification of whole-cell BATF2 (*J*) and cyclin D1 (*K*) protein levels of human astrocytes and U87-MG cells shown in *I*, normalized to vinculin expression. Data points are representative of replicates from two independent experiments. The bars represent mean ± SEM. BATF2, basic leucine zipper ATF-like transcription factor 2; DAPI, 4′,6-diamidino-2-phenylindole.

To determine how cell cycle genes were regulated by BATF2, media-treated wildtype or *Batf2*^−/−^ murine astrocytes were cultured, and the levels of *Cks1b*, C*dk2*, and *Ccnd1* transcript were quantified. Importantly, loss of BATF2 resulted in increased expression of these target cell cycle genes (Fig. 3, *A*–*C*). To confirm this increase was specific to intrinsic BATF2 expression and not an artifact of the *Batf2*^−/−^ environment during astrocyte development, we knocked down *BATF2* in wildtype human astrocytes using siRNA and observed similar changes in gene signature (Fig. S2, *A*–*D*). Further, protein upregulation of cyclin D1 in *Batf2*^−/−^ astrocytes compared with controls was confirmed *via* Western blot (Fig. 3, *D* and *E*) and immunocytochemistry (Fig. 3, *F*–*I*), along with protein levels of CDK2 and CKS1B (Fig. S3). U87-MG glioma cells were then used as a model of BATF2 overexpression for reciprocal analysis of BATF2-regulated cell cycle genes. Upregulated *BATF2* transcript was confirmed in U87-MG cells compared with human astrocytes, and subsequent downregulation of *CCND1*, *CKS1B*, and *CDK2* gene expression was observed (Fig. 4, *A*–*D*). Overexpression of BATF2 protein was demonstrated in U87-MG cells *via* immunocytochemistry and Western blot (Fig. 4, *E*–*J*). Interestingly, this correlated with a decrease in cyclin D1 protein compared with human astrocytes in a similar manner to transcript levels (Fig. 4, *I* and *K*). Together, this suggests that BATF2 targeting of cell cycle motifs has a functional outcome of reducing cell cycle machinery at the protein level.

### BATF2 regulates cyclin D1 in two separate models of aberrant proliferation

**Figure 5:**
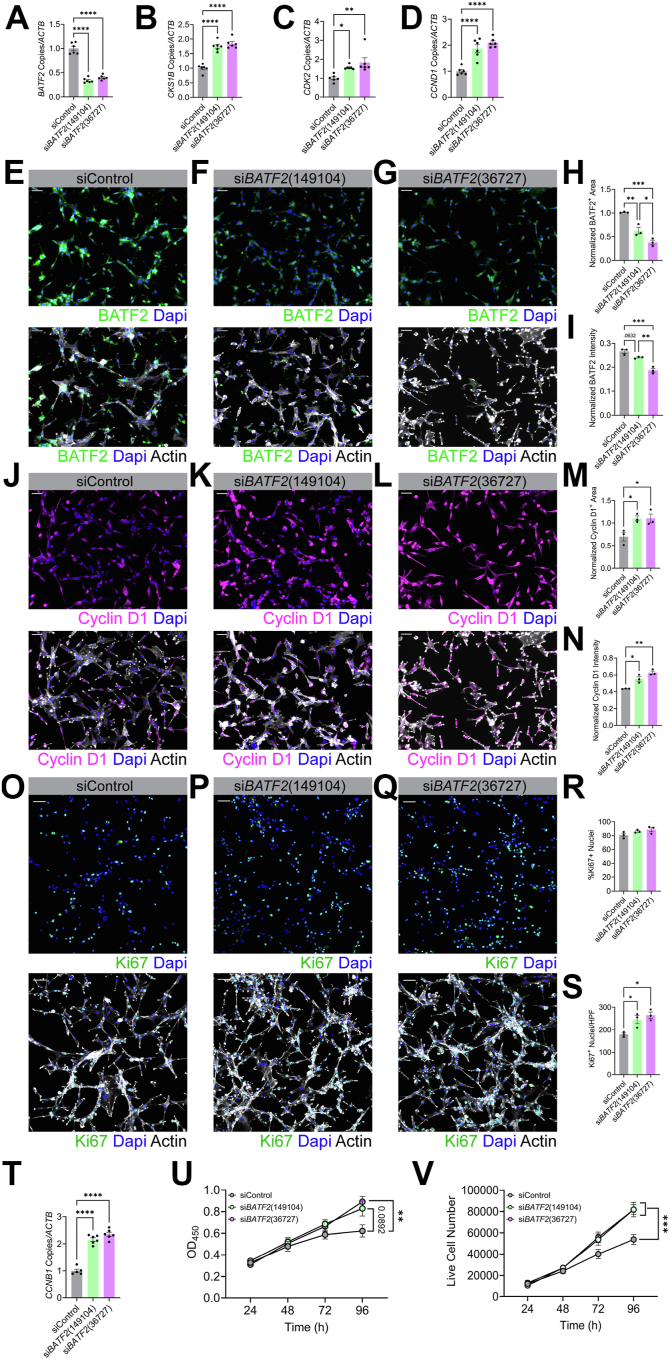
**Knockdown of *BATF2* upregulates cell cycle genes in U87-MG cells.***A–D,* quantification of *BATF2* (*A*), *CKS1B* (*B*), *CDK2* (*C*), and *CCND1* (*D*) gene expression in U87-MG cells treated with siControl or si*BATF2* for 72 h. Data points were normalized to the siControl average and are representative of replicates from two independent experiments. ∗*p* < 0.05, ∗∗*p* < 0.01, ∗∗∗∗*p* < 0.0001 compared with siControl samples by one-way ANOVA. The bars represent mean ± SEM. *E–G,* U87-MG cells treated with siControl (*E*) and si*BATF2* (*F* and *G*) labeled for BATF2, actin, and nuclei counterstained with DAPI. The scale bars represent 50 μm. *H* and *I,* quantification of BATF2-positive area (*H*) and intensity (*I*) for U87-MG cells treated with siControl or si*BATF2*. Data points were normalized to actin expression and are representative of technical replicates. ∗*p* < 0.05, ∗∗*p* < 0.01, ∗∗∗*p* < 0.001 compared with siControl samples by one-way ANOVA. The bars represent mean ± SEM. *J–L,* U87-MG cells treated with siControl (*J*) and si*BATF2* (*K* and L) labeled for cyclin D1, actin, and nuclei counterstained with DAPI. The scale bars represent 50 μm. *M* and *N,* quantification of cyclin D1-positive area (*M*) and intensity (*N*) for U87-MG cells treated with siControl or si*BATF2*. Data points were normalized to actin expression and are representative of technical replicates. ∗*p* < 0.05, ∗∗*p* < 0.01 compared with siControl samples by one-way ANOVA. *O–Q,* U87-MG cells treated with siControl (*O*) and si*BATF2* (*P* and *Q*) labeled for Ki67, actin, and nuclei counterstained with DAPI. The scale bars represent 50 μm. *R* and *S,* quantification of Ki67-positive nuclei for siControl or si*BATF2*-treated U87-MG cells. Data points in *R* were normalized to the total nuclei count. Data shown in *R* and *S* are representative of technical replicates. ∗*p* < 0.05 compared with siControl samples by one-way ANOVA. The bars represent mean ± SEM. *T,* quantification of *CCNB1* transcript expression in siControl or si*BATF2*-treated U87-MG cells. Data points were normalized to the siControl average and are representative of replicates from two independent experiments. ∗∗∗∗*p* < 0.0001 compared with siControl samples by one-way ANOVA. The bars represent mean ± SEM. *U,* cell viability assay of siControl or si*BATF2-*treated U87-MG cells over 96 h. Data points are representative of the combined average of absorbance values at 450 nm from individual replicates from two independent experiments (siControl *N* = 4–6, si*BATF2* (149104) *N* = 5–6, si*BATF2* (36727) *N* = 4 to 6 per timepoint). ∗∗*p* < 0.01 compared with siControl samples by one-way ANOVA. The bars represent mean ± SEM. *V,* live cell counts for siControl or si*BATF2*-treated U87-MG cells over 96 h. Data points are representative of the combined average of live cell counts of individual replicates from two independent experiments. (siControl *N* = 4, si*BATF2* (149104) *N* = 3–4, si*BATF2* (36727) *N* = 4 per timepoint). ∗∗∗*p* < 0.001 compared with siControl samples by one-way ANOVA. The bars represent mean ± SEM. BATF2, basic leucine zipper ATF-like transcription factor 2; DAPI, 4′,6-diamidino-2-phenylindole.

siRNA-targeted silencing of *BATF2* in U87-MG cells was then employed to determine if knockdown of BATF2 expression was sufficient to modulate cell cycle gene expression. Downregulation of *BATF2* transcript was confirmed, as we demonstrated a greater than 50% knockdown efficiency compared with controls (Fig. 5*A*). Levels of *CKS1B*, *CDK2*, and *CCND1* transcripts were quantified and found to be upregulated in cells with *BATF2* knockdown compared with negative controls (Fig. 5, *B*–*D*). Moreover, reduction of BATF2 protein was confirmed in si*BATF2-*treated cells using immunocytochemical staining (Fig. 5, *E*–*I*), and upregulation of cyclin D1 protein levels was demonstrated in conjunction with decreased BATF2 (Fig. 5, *J*–*N*). To evaluate the impact of *BATF2* knockdown on proliferation, Ki67 expression was assessed. Although the percentage of Ki67^+^ nuclei remained unchanged, the total number of Ki67^+^ cells per high-powered field increased in *BATF2* knockdown groups, indicating a higher cell density (Fig. 5, *O*–*S*). Further, additional markers of proliferation, such as cyclin B1, were transcriptionally upregulated following knockdown of *BATF2* (Fig. 5*T*). Cell viability and live cell count were also increased over time compared with scramble controls (Fig. 5, *U* and *V*). Collectively, these data demonstrate that BATF2 functions to suppress genes associated with cell cycle progression, and its loss leads to increased proliferative activity.

**Figure 6:**
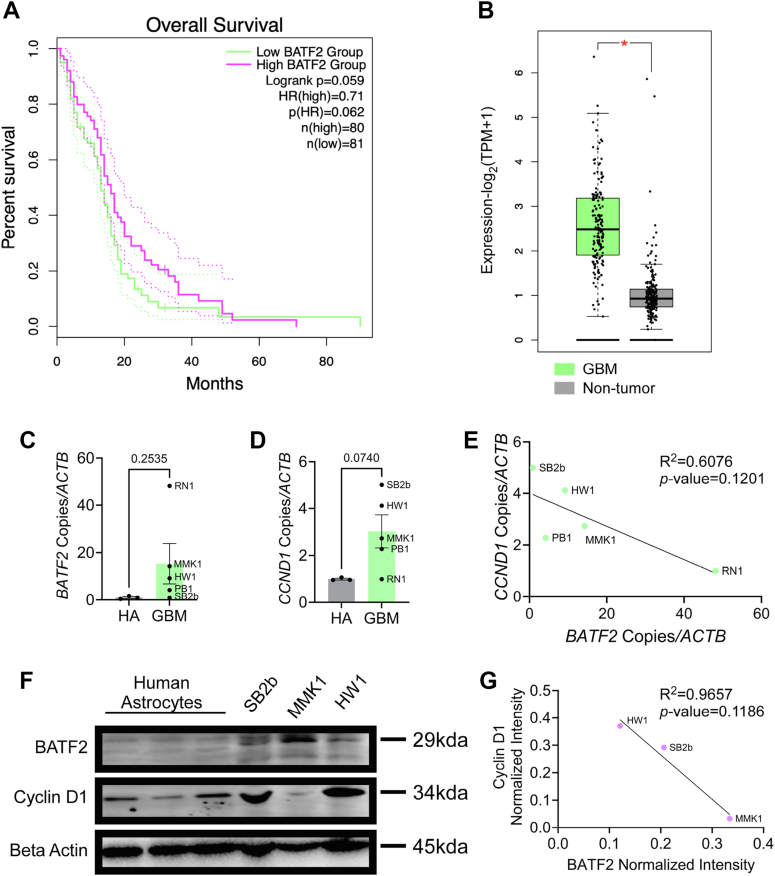
**BATF2 expression negatively correlates with cyclin D1 levels in GBM.***A,* overall survival outcomes relative to *BATF2* expression in GBM patients. *B,* quantification of *BATF2* expression between GBM and nontumor samples (GBM *N* = 163, nontumor *N* = 207). Data represented in *A* and *B* were obtained from TCGA and GTEx publicly available data sets and were analyzed using Gepia2. ∗*p* < 0.05 compared with nontumor samples. The bars represent mean ± SD. *C* and *D,* quantification of *BATF2* (*C*) and *CCND1* (*D*) gene expression in human astrocytes and GBM patient samples. Data points were normalized to the human astrocyte average. Human astrocyte data points are representative of technical replicates. GBM data points are representative of individual patients. The bars represent mean ± SEM. *E,* regression analysis of *CCND1 versus BATF2* gene expression in GBM patients. Data points were analyzed by simple linear regression and are representative of individual patients. *F,* representative Western blot of whole-cell BATF2, cyclin D1, and beta-actin protein levels in human astrocytes and GBM patient samples. *G,* regression analysis of cyclin D1 versus BATF2 protein expression in GBM patients. Data points were analyzed by simple linear regression and are representative of individual patients. BATF2, basic leucine zipper ATF-like transcription factor 2; GBM, glioblastoma multiforme; GTEx, Genotype-Tissue Expression; TCGA, The Cancer Genome Atlas.

Given the demonstrated inverse relationship between BATF2 and cyclin D1 exhibited in both models of BATF2 deficiency and overexpression, we asked if a similar dynamic may also exist in a CNS model of aberrant proliferation. Initially, we examined how *BATF2* expression affected survival in GBM patients using the publicly available The Cancer Genome Atlas-GBM dataset and found that higher *BATF2* transcript correlated with increased survival (Fig. 6*A*). We also observed an overall increase in *BATF2* expression in GBM patients compared with nontumor control samples, correlating with our findings in U87 cells (Fig. 6*B*). Moreover, we also found that GBM tumor type affected survival with regard to *BATF2* expression, with classical and proneural forms demonstrating the greatest increase in survival (Fig. S4). To test the relationship between BATF2 and cyclin D1, we examined *BATF2* and *CCND1* transcript expression across several GBM patient samples (42) and found that they both tended to be upregulated in comparison with human astrocyte controls (Fig. 6, *C* and *D*). Intriguingly, regression analysis of *CCND1* and *BATF2* demonstrated an inverse correlation, in which patients with higher *BATF2* levels expressed lower *CCND1* levels (Fig. 6*E*). This trend was also demonstrated at the protein level, with a higher coefficient of fitness compared with transcript (Fig. 5, *F* and *G*), suggesting that BATF2 suppression of cyclin D1 may be sustained throughout the disease.

## Discussion

To our knowledge, this is the first study to demonstrate that BATF2 directly binds to cell cycle genes in non-neoplastic cells. These findings suggest that BATF2 may work to prevent the aberrant proliferation of astrocytes within the context of CNS homeostasis by counteracting the overexpression of cell cycle proteins, including CKS1B, CDK2, and cyclin D1. Notably, the downregulation of cell cycle–stimulating proteins, such as CDK2, is necessary to drive differentiation of neural stem cell populations, and consistent expression maintains proliferation and stem cell–like properties (43, 44, 45, 46). Overexpression of the CDK4–cyclin D1 complex has also demonstrated similar effects, in that it prevents the genesis of neural stem cell populations and instead promotes the expansion of basal progenitors (47, 48). While proliferation is necessary in particular brain regions, such as the cortical subventricular zones that may require constitutive CDK2 and cyclin D1 expression to sustain progenitor populations, cell cycle progression is restricted in other postnatal CNS compartments. As a result, BATF2 expression may contribute to the maintenance of the differentiated state of mature CNS cells and function to moderate cell division in specific contexts.

In addition, recent evidence implies a role for BATF2 in regulating the cell cycle and proliferation in multiple cancer models. Specifically, indirect regulation of cyclin D1 by BATF2 has been characterized in colorectal cancer, in which sequestration of BATF2 in the cytosol *via* interaction with the nuclear export sequence of chromosome region maintenance 1 contributes to increased cellular proliferation through heightened activation of the AP-1–cyclin D1–phosphoretinoblastoma signaling axis (37). In addition, in gastric cancer, BATF2 has been shown to complex with tumor protein p53 to suppress extracellular signal–regulated kinase signaling and subsequent induction of cyclin D1 expression (38). Here, we add an additional layer to the known functions of BATF2 as a cell cycle regulator, suggesting that BATF2 suppression of *CCND1*, along with other cell cycle genes, may also occur at the transcriptional level in addition to these previously described protein-to-protein interactions. Future analyses using luciferase reporter assays and electrophoretic mobility shift assays would further complement our ChIP-Seq data and strengthen the evidence that BATF2 regulates cell cycle gene expression at the transcriptional level.

Furthermore, in the context of CNS cancers like glioblastoma, BATF2 expression has been shown to limit tumor growth primarily by altering the tumor microenvironment with minimal impact on the proliferation of glioma cells, as indicated by analysis of proliferating cell nuclear antigen, a common marker of DNA replication and proliferation (39). In this study, we demonstrate that loss of BATF2 does increase the expression of proliferation markers in *Batf2*^−/−^ astrocytes; however, this effect was observed in otherwise normal cells that lack many of the genetic mutations detected in glioblastoma (49, 50). Therefore, we also assessed BATF2 and cell cycle gene expression in GBM patient samples (42). We found that high levels of BATF2 correlated with increased survival and that overall *BATF2* transcript levels were increased in GBM samples compared with controls. Further, we found that high BATF2 levels correlated with lower cyclin D1 levels at both transcript and protein levels. While these data suggest that BATF2 may be upregulated as a compensatory mechanism to regulate cyclin D1 in GBM, overall cyclin D1 levels were still elevated in GBM samples compared with human astrocyte controls. In addition, survival outcomes differed by GBM tumor subtype, with minimal effects observed in mesenchymal and neural forms, indicating that additional genetic variations present across subtypes of GBM impact the efficacy of BATF2 as a tumor suppressor. This suggests that increased proliferation likely still occurs in GBM despite BATF2 upregulation and that BATF2 alone is not sufficient to mitigate abnormal cell division during GBM. As a result, further studies using larger patient cohorts are required to fully elucidate how BATF2 may modulate proliferation in this context.

Astrocyte proliferation occurs locally in response to inflammatory stimuli. We have recently demonstrated that BATF2 is upregulated in astrocytes downstream of interferon-γ during neuroinflammation. Further, we found that BATF2 is expressed in the nucleus of astrocytes in the core of multiple sclerosis lesions (35), where astrocytes proliferate and participate in glial scar formation. Notably, upregulation and activation of proproliferative molecules such as AP-1 subunits have been cited in other glial cell populations in multiple sclerosis lesions (51). In addition, in animal models of CNS demyelination, downregulation of cell cycle regulatory genes, like CDK2, in oligodendrocyte progenitor cells has been shown to accelerate remyelination through promoting cell cycle exit (52). As a result, the upregulation of BATF2 in response to inflammation in astrocytes may function to regulate proper proliferation to promote reparative processes; however, whether this process may occur specifically through BATF2 interaction with AP-1 proteins or at the transcriptional level requires further investigation.

## Experimental procedures

### Animals

*Batf2*^*−/−*^ mice (strain no.: 031903) were obtained from The Jackson Laboratories. Mice were bred as heterozygous pairs, and wildtype and homozygous knockout littermates of both sexes were used for subsequent experiments. All housing, breeding, and procedures were approved by the Institutional Animal Care and Use Committee at the Lerner Research Institute, Cleveland Clinic Foundation (Cleveland, OH) using protocol number 1862. All mice used were on a C57BL/6NJ background, maintained on a 14/10 h light–dark cycle, and had *ad libitum* access to food and water. Mice (housed 2–5/cage) did not have any prior history of drug administration, surgery, or behavioral testing.

### Cell culture

Primary human astrocytes were obtained commercially from ScienCell Laboratories and grown according to provided protocols in complete ScienCell Astrocyte Medium. Briefly, isolated astrocytes were assessed for morphology by phase and relief contrast microscopy and glial fibrillary acidic protein positivity by immunofluorescence. Cell number, viability (≥70%), proliferative potential (≥15 pd), and negative screening for potential biological contaminants and mycoplasma were confirmed by ScienCell prior to cryopreservation. Following receipt, astrocytes were passaged, and P3 cells were used for all studies. Quantitative PCR analysis for astrocyte purity was conducted prior to use in all experiments.

Primary murine astrocytes were harvested as previously described (53) with minor modifications. P2–P4 wildtype and *Batf2*^−/−^ pups were euthanized, and CNS tissue was digested with gentle agitation for 15 min in 0.25% trypsin–EDTA. Trypsinization was stopped by adding Dulbecco's modified Eagle's medium (DMEM) supplemented with 10% heat-inactivated fetal bovine serum and 1% penicillin–streptomycin (10% DMEM). Cells were then treated with 50 μg/ml of DNase I for 3 min. Single-cell suspensions were then made *via* trituration, and cells were pelleted *via* centrifugation at 500*g* for 5 min. Cells were resuspended in 10% DMEM and plated onto fibronectin-coated plates. Cells were incubated at 37 °C and 5% CO_2_ and received a complete media change every other day until reaching 80% confluency. Astrocytes were mechanically separated from microglia and oligodendrocyte progenitor cells *via* orbital shaking.

U87-MG cells were obtained from the American Type Culture Collection and cultured at 37 °C and 5% CO_2_ in Eagle's minimum essential medium containing 10% fetal bovine serum and 1% penicillin–streptomycin. Cells were used within 12 passages for all experiments.

### Cell viability and live cell counts

Murine astrocytes and U87-MG cells were plated into 96-well plates, and cell viability was determined daily for a period of 4 days (96 h) using a Cell Counting Kit-8 (Bio-Techne; catalog no.: 7368). Absorbances (450 nm) were recorded daily using a Multiskan FC microplate reader (ThermoFisher). Corresponding live cell counts were determined from murine astrocytes and U87-MG cells plated into 24-well plates. Cells were counted on a Countess II automated cell counter daily over 4 days (96 h) using Trypan Blue exclusion dye (ThermoFisher; catalog no.: 15250061).

### siRNA knockdown

Silencer predesigned BATF2 siRNAs (AM16708, 149104, and 36727) and negative control siRNA (AM14641) were purchased from Invitrogen. U87-MG cells were harvested at roughly 80% confluency using 0.05% trypsin–EDTA. Cells were then prepared for nucleofection using an Amaxa SE Cell Line 4D Nucleofection XL Kit (V4XC-1024) according to the manufacturer's protocol. Cells/cuvette (1 × 10^6^) were transfected at a time. siRNA or negative control (5 ng/μl) was added to each designated cuvette, and uptake was facilitated using a Lonza 4D Nucleofector Core Unit using pulse code DS-126. Immediately following nucleofection, cells were cultured for 72 h prior to protein or RNA collection.

### Quantitative PCR

Total RNA was collected using an RNeasy Mini Kit (Qiagen) according to the manufacturer's instructions and measured with a NanoDrop One spectrophotometer (ThermoFisher). Total RNA was then normalized, treated with DNase I (ThermoFisher), and reverse transcribed to complementary DNA using a TaqMan Reverse Transcription kit (Applied Biosciences). Prepared complementary DNA was then used as a template for quantitative PCR using a QuantStudio 6 Real-Time PCR system (ThermoFisher). Relative gene expression was quantified after normalizing to *GAPDH* or *ACTB* expression. Primer sequences are listed in Table S1.

### Western blotting

Cells were trypsonized with 0.05% (human) or 0.25% (murine) trypsin–EDTA and homogenized in immunoprecipitation lysis buffer (50 mM Tris–HCl [pH = 7.4], 150 mM NaCl, 1 mM EDTA, 1% Triton X-100, and 1:100 proteinase/phosphatase inhibitor). Samples were briefly triturated, vortexed for 3 s, and incubated on ice for 30 min. For complete lysis, samples were frozen on dry ice for 3 min and thawed in wet ice for 15 min. A total of three freeze–thaw cycles were conducted. Samples were then centrifuged at 13,000*g* for 30 min, and supernatants were collected for immunoblotting. Protein levels were quantified using a Pierce BCA kit (ThermoFisher) and normalized. Laemmli sample buffer (4x) and β-mercaptoethanol were added to normalized samples. The total protein lysate (10–20 μg) was resolved on Novex WedgeWell 4% to 20% Tris–glycine gels and transferred to polyvinylidene difluoride membranes. The membranes were blocked with 3% bovine serum albumin in Tris-buffered saline and 0.1% Tween-20 (TBST) for 1 h at room temperature and incubated overnight at 4 °C with primary antibody. Membranes were blotted with primary antibodies for BATF2 (Invitrogen; PA5-37138), cyclin D1 (Santa Cruz, SC717 and Proteintech, 60186-I-Ig), CDK2 (78B2) (Cell Signaling, 2546S), cyclin B1 (D5C10) (Cell Signaling, 12231S), and vinculin (Invitrogen, 700062). The following day, membranes were washed with TBST three times and incubated with horseradish peroxidase–conjugated antibodies for 1 h at room temperature. Membranes were then washed with TBST three times and imaged using the ChemiDoc MP imaging system (Bio-Rad) after activation with ECL substrate (Bio-Rad). Uncropped blots are demonstrated in Figure S5.

Glioblastoma patient samples were derived from the study by Stringer *et al.* (42) and human astrocytes used for comparison were prepared as described previously and transferred onto low fluorescence polyvinylidene difluoride membranes following gel resolution. Blots were blocked in 3% bovine serum albumin in TBST for 1 h at room temperature and incubated with primary antibodies for BATF2 (Invitrogen; PA5-37138) and cyclin D1 (Proteintech; 60186-I-Ig) overnight at 4 °C. The following day, the blot was washed with TBST three times and incubated with fluorescently conjugated secondary antibodies for 1 h at room temperature and imaged on an ODYSSEY CLx LI-COR system using Image Studio v5.2. The blot was then incubated in stripping buffer (15 g glycine, 10 ml of 10% SDS, 10 ml Tween 20/1000 l, pH adjusted to 2.2) for two 10-min periods. The blots were then incubated in PBS for two 10-min periods followed by two 5-min washes in TBST. Membranes were then blocked and probed with a primary antibody for Beta Actin (ThermoFisher; MA5-15739) for 1 h at room temperature. The blot was then washed three times with TBST and incubated with a horseradish peroxidase–conjugated antibody for 1 h at room temperature. The membrane was then washed three times with TBST and imaged using the ChemiDoc MP imaging system (Bio-Rad) after activation with ECL substrate. Protein intensities were quantified using ImageJ software (v1.52a, NIH) and normalized to loading controls. Uncropped blots are demonstrated in Figure S6.

### Immunocytochemistry

Cells were plated into chamber slides and allowed to grow until roughly 70% confluency. Cells were then fixed with 4% paraformaldehyde in PBS for two 10-min periods and washed three times with PBS for 5 min. Slides were blocked with 10% goat serum (Sigma) and 0.3% Triton X-100 for 15 min at room temperature and then incubated with primary antibodies for BATF2 (Santa Cruz; SC293274), cyclin D1 (Santa Cruz; SC717), phospho-histone H3 (Ser10) (Cell Signaling; 9701S), Ki67 (Abcam; AB15580), pMCM2 (Ser139) (D1Z8X) (Cell Signaling; 12958S), and CKS1B (Invitrogen; 36-6800) overnight at 4 °C. The following day, slides were washed three times with PBS for 5 min and probed with Alexa Fluor 488 and 647 secondary antibodies for 15 min at room temperature. Actin was then counterstained using an ActinRed 555 Ready Probe (Invitrogen; R37112), and nuclei were counterstained with 4′,6-diamidino-2-phenylindole for 10 min at room temperature, each diluted in PBS. Slides were imaged using the 10x objective of a confocal microscope LSM 800 (Carl Zeiss) or the 20x objective of a Keyence BZ-X710 microscope. Representative images shown are illustrative of 4 to 10 images per individual well of a chamber slide for human samples and per individual animal for murine samples. The mean positive area, mean intensity, Mander's coefficient of colocalization, and percent positive nuclei were determined by setting thresholds using appropriate controls and quantified using ImageJ (version 1.52a) and Fiji (version 1.54f) software. Each of these measurements was calculated per image and then averaged across individual wells for human samples and individual animals for murine samples to represent data points depicted.

### ChIP-Seq and pathway analysis

ChIP-Seq of human astrocytes was previously conducted and reported (35). Genes of media-treated astrocytes were analyzed using IPA software, version 111725566. Pathways reported in Figure 2 are representative of the top five molecular mechanisms and diseases associated with BATF2-bound genes. A full list of molecular mechanisms and diseases identified by IPA is provided as an attached file to this article.

### Survival and BATF2 GBM expression analysis

Gene expression and survival data for GBM patient samples in Figure 6, *A* and *B* were obtained from The Cancer Genome Atlas (54, 55) and Genotype-Tissue Expression (56, 57) projects and analyzed using GEPIA2 (58).

### Statistical analysis

Data and statistical analysis were performed using Prism 10.1.2 (GraphPad). Significance criteria included ∗*p* < 0.05, ∗∗*p* < 0.01, ∗∗∗*p* < 0.001, and ∗∗∗∗*p* < 0.0001. All statistical analyses were conducted using a two-tailed Student's *t* test or one-way ANOVA. Correlation analyses were conducted using a standard linear regression. Bar graphs indicate mean ± SEM unless otherwise noted in the figure legend. For human astrocyte and U87-MG cell analysis, each data point is representative of an individual technical replicate. For murine samples, each data point is representative of an individual animal. For GBM samples, each data point is representative of an individual patient. All experiments were conducted independently at least twice.

## Data availability

ChIP-Seq data have been deposited in Gene Expression Omnibus with accession number GSE288440. All remaining data supporting the reported findings are available from the corresponding author upon reasonable request.

## Supporting information

This article contains supporting information.

## Conflict of interest

The authors declare that they have no conflicts of interest with the contents of this article.

## Supporting information

Supplemental Figures

IPA Diseases and Functions
