# Supplementary material for: BATF2-mediated control of astrocyte proliferation
Source: J Biol Chem. 2025 Sep 12;301(11):110710. doi: 10.1016/j.jbc.2025.110710 (PMC12605025; doi:10.1016/j.jbc.2025.110710)
Supplement: Supplemental Figures [file mmc1.pdf]

Supplemental Figures

Table S1. List of Primers

Gene Name	Forward Sequence	Reverse Sequence	Reference
Human *GAPDH*	GAAGGTGAAGGTCGGAGTC	GAAGATGGTGATGGGATTTC	(59, 60)
Human *ACTB*	ACCTTCTACAATGAGCTGCG	CCTGGATAGCAACGTACATGG	(61)
Human *BATF2*	AGACCCCAAGGAGCAACA	CAGGGCGAGGTTGTCTTT	(62)
Human *CKS1B*	AGGAATCTTGGCGTTCAGCA	TGGTTTCTTGGGTAGTGGGC	This paper
Human *CDK2*	GCATTCCTCTTCCCCTCATCA	GGTCCCCAGAGTCCGAAAGA	This paper
Human *CCND1*	ATGCCAACCTCCTCAACGAC	AAGACCTCCTCCTCGCACTT	This paper
Human *CCNB1*	AGCCAGAACCTGAGCCTGTTA	ATTGGGCTTGGAGAGGCAGT	This paper
Murine *Gapdh*	GGCAAATTCAACGGCACAGT	AGATGGTGATGGGCTTCCC	(59, 60)
Murine *Batf2*	GCCCAGCGCAGCCGGCAGAA	CCAGCTCAGTCTGCAAGGCCT	(35)
Murine *Cks1b*	AGGAATCCACCGAAGCTGGT	CAGTGTAGCTCCCAAGTGGC	This paper
Murine *Cdk2*	ACCGAGCACCTGAAATTCTTCT	TCCTTGTGATGCAGCCACTTC	This paper
Murine *Ccnd1*	AAAATGCCAGAGGCGGATGA	GAAAGTGCGTTGTGCGGTAG	This paper
Murine *Ccnb1*	TAATCCCTCTCCAAGCCCGA	TCACTTCACGACCCTGTAGGT	This paper

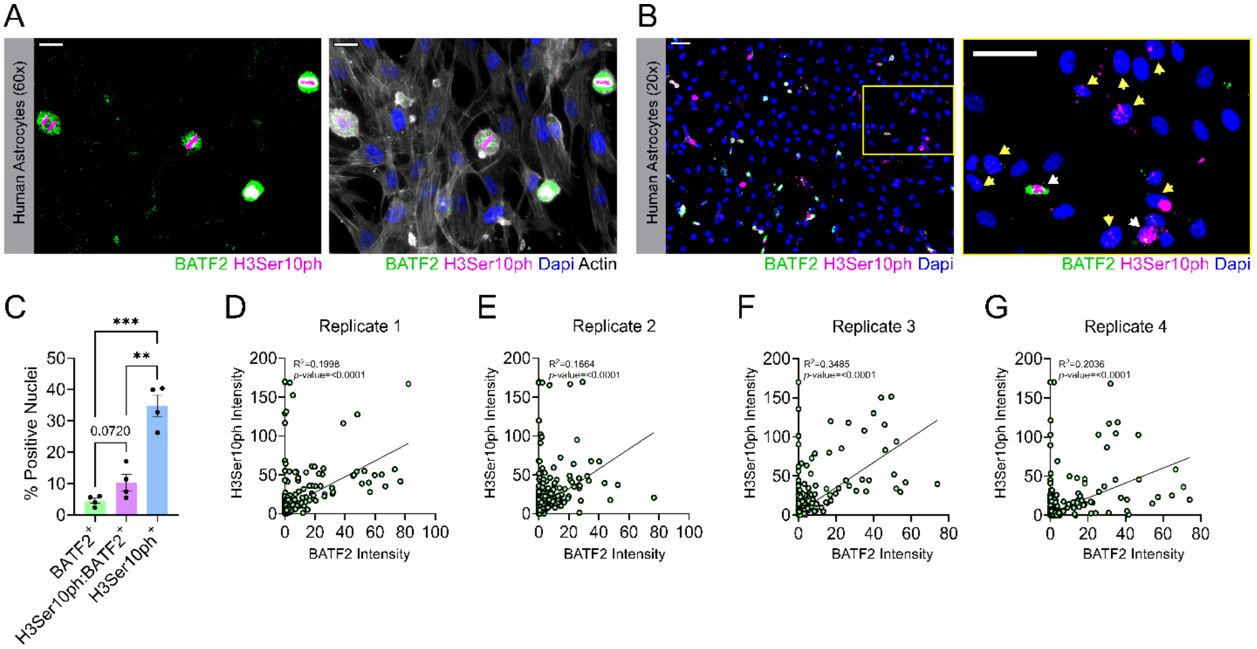

Supplemental Figure 1. BATF2 is expressed in mitotic cells.

A, 60x images of human astrocytes labeled for BATF2, H3Ser10ph, actin, and nuclei counterstained with Dapi. Scale bars, 10 μm. *B*, 20x image and digital zoom inset of human astrocytes labeled for BATF2, H3Ser10ph, actin, and nuclei counterstained with Dapi. Yellow arrows indicate H3Ser10ph single positive nuclei and white arrows indicate BATF2:H3Ser10ph double positive nuclei. Scale bars, 50 μm. *C*, quantification of BATF2 single positive, BATF2:H3Ser10ph double positive, and H3Ser10ph single positive nuclei. Data points were normalized to the total nuclei count and are representative of individual technical replicates. ***p* <0.01, ****p* <0.001 by one-way ANOVA. *D-G*, correlation plots of H3Ser10ph and BATF2 intensity over 4 individual replicate experiments. Data points are representative of individual cells.

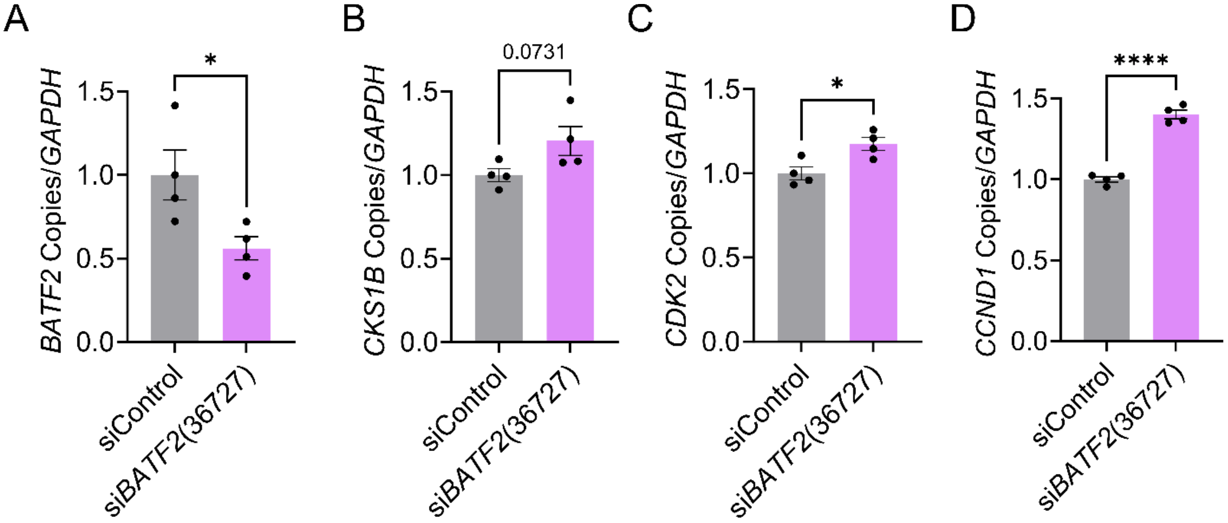

Supplemental Figure 2. *BATF2* knockdown in human astrocytes increases cell cycle gene expression. *A-D*, quantification of *BATF2* (A), *CKS1B* (B), *CDK2* (C), and *CCND1* (D) gene expression in human astrocytes cells treated with siControl or si*BATF2*(36727) for 72 h. Data points were normalized to the siControl average and are representative of replicates from two independent experiments. *p <0.05, ***p <0.001 compared to siControl samples by Student's t test. Bars represent mean ± SEM.

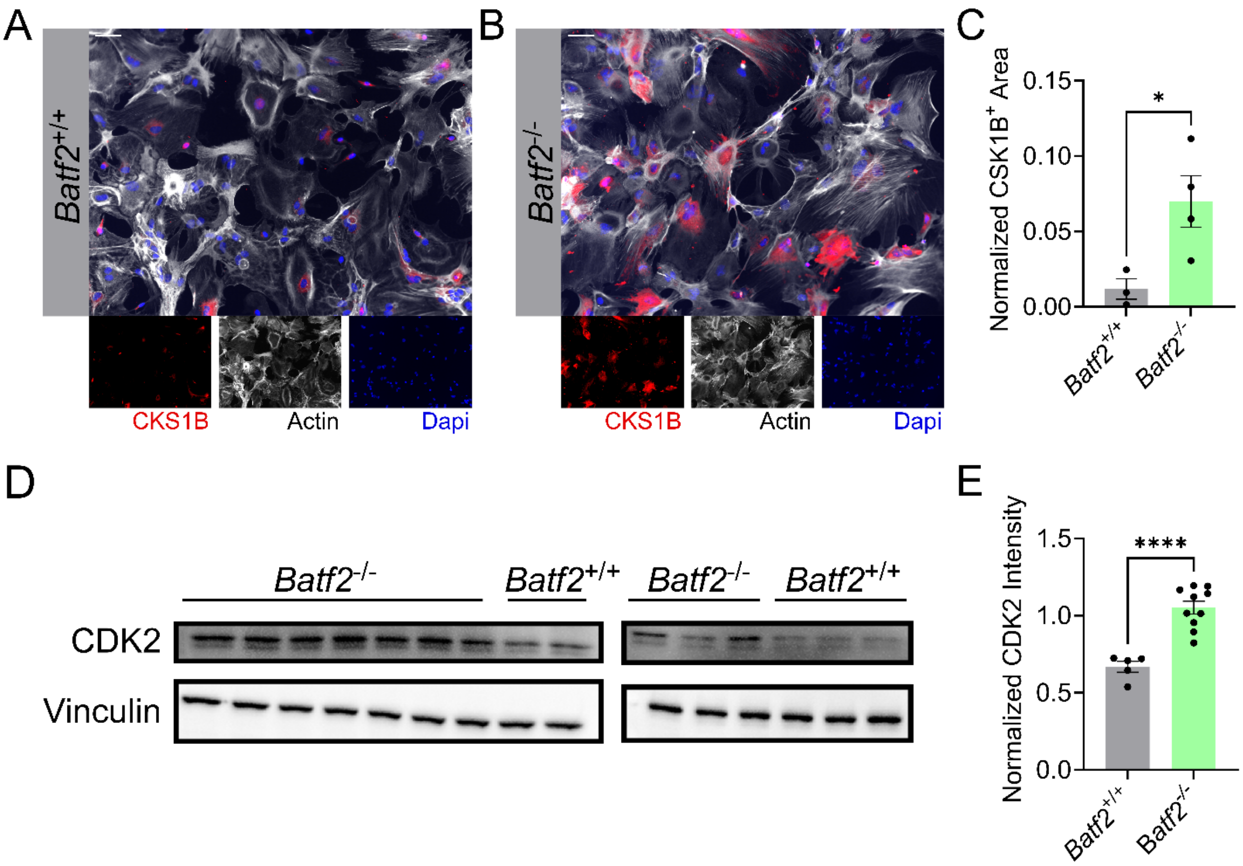

Supplemental Figure 3. CKS1B and CDK2 protein levels are upregulated in *Batf2*$^{-/-}$ astrocytes. *A-B*, wildtype (A) and *Batf2*$^{-/-}$ (B) astrocytes labeled for CKS1B, actin, and nuclei counterstained with Dapi. Scale bars, 50 µm. *C*, quantification of CSK1B positive area for wildtype and *Batf2*$^{-/-}$ mice. Data were normalized to actin area and datapoints are representative of individual mice. *$p < 0.05$ compared to wildtype samples by two-tailed Student's *t* test. Bars represent mean ± SEM. *D,* representative Western blot of whole cell CKS1B and vinculin protein levels in wildtype and *Batf2*$^{-/-}$ astrocytes. *E,* quantification of whole cell CKS1B protein levels in wildtype and *Batf2*$^{-/-}$ astrocytes shown in D, normalized to vinculin expression. Data points are representative of individual mice. ****p <0.0001 compared to wildtype by Student's *t* test. Bars represent mean ± SEM.

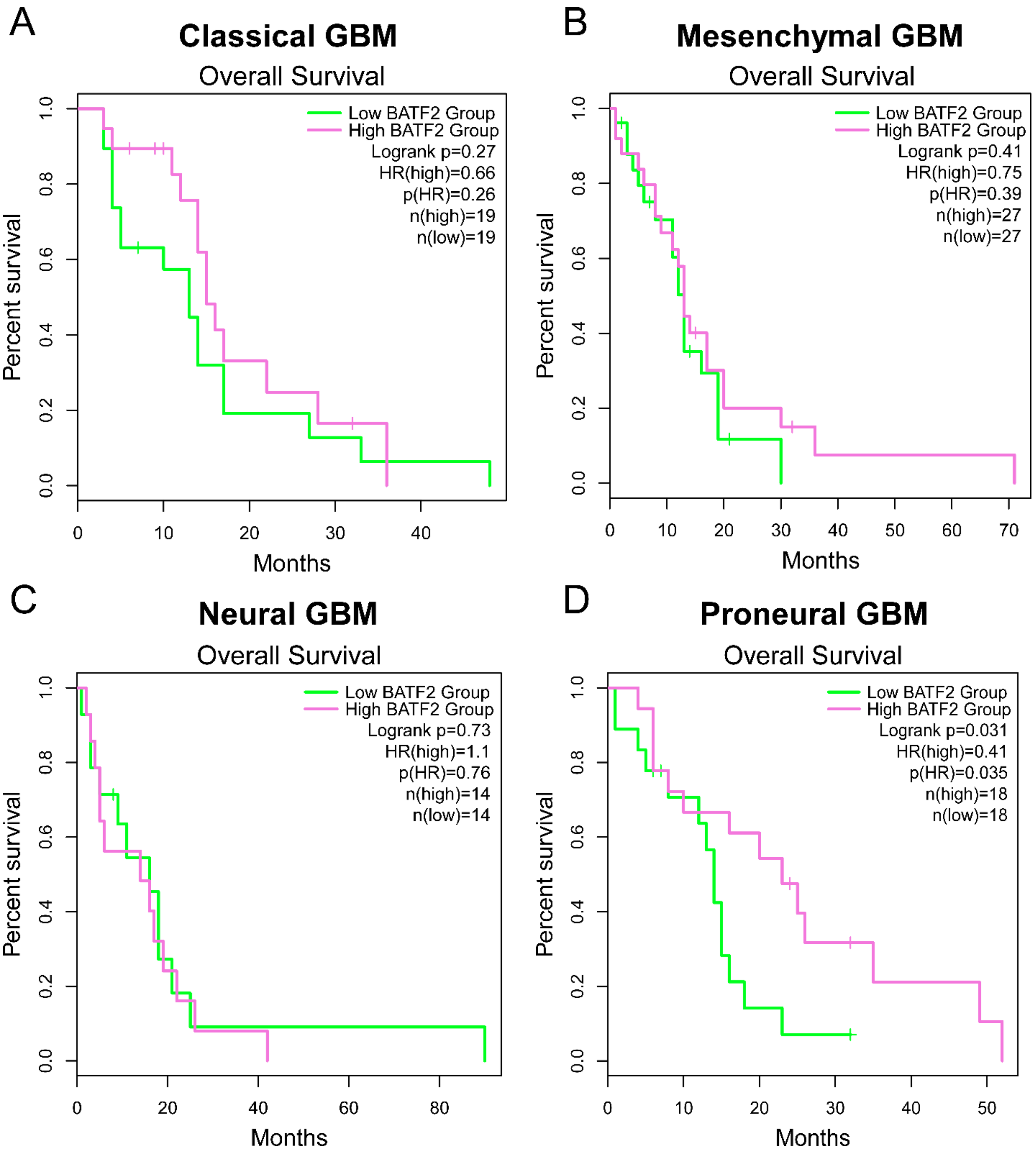

Supplemental Figure 4. GBM tumor sub-type survival association with *BATF2* expression. *A-D*, survival analysis of *BATF2* expression in GBM patients with classical (A), mesenchymal (B), neural (C), and proneural (D) subtypes. Data represented in A-D were obtained from TCGA and GTEx publicly available data sets and were analyzed using Gepia2.

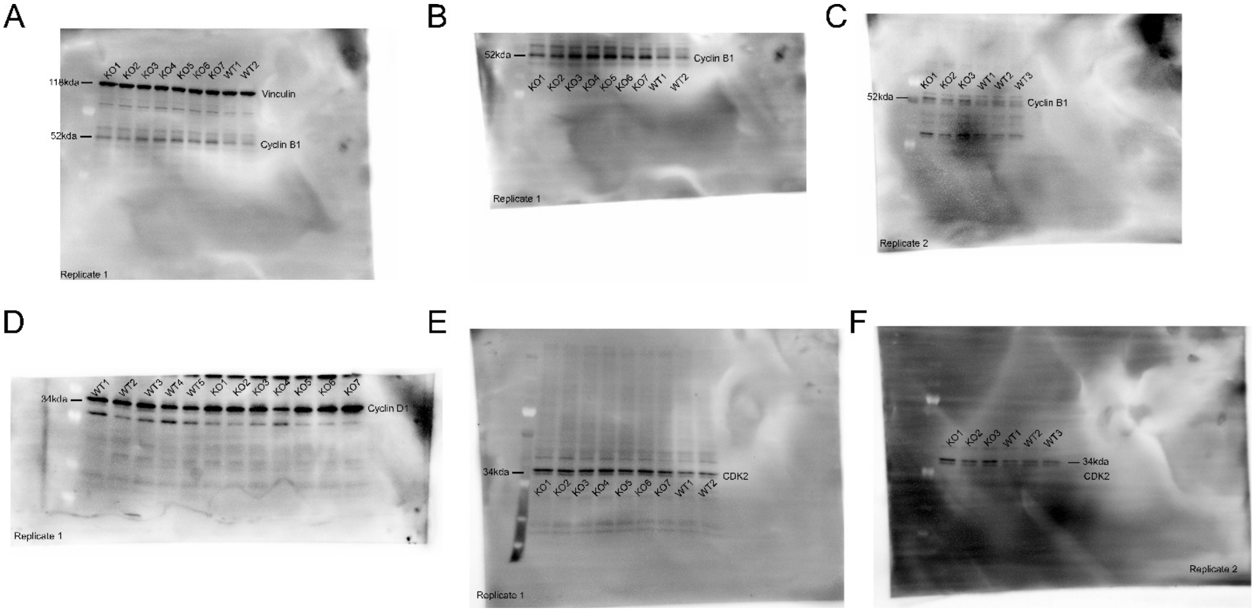

Supplemental Figure 5. Uncropped images of Western blots from murine astrocyte protein.

A-F, Representative Western blots of whole cell cyclin B1 and vinculin (A), cyclin B1 (B-C), cyclin D1 (D), and CDK2 (E-F) levels in wildtype and *Batf2*$^{-/-}$ astrocytes.

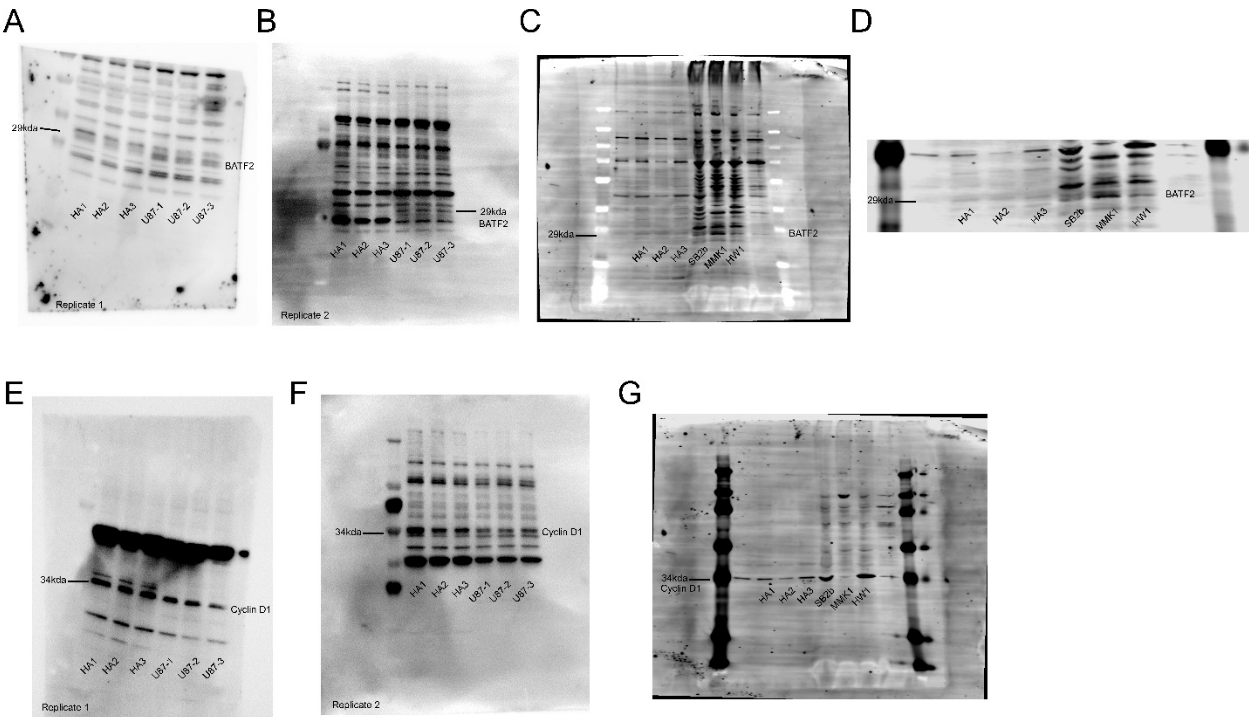

Supplemental Figure 6. Uncropped images of Western blots from human protein Western blots. *A,B,E,F*, Representative Western blots of whole cell BATF2 (A-B) and cyclin D1 (E-F) in human astrocytes and U87-MG cells. *C,D,G,* Representative Western blots of whole cell BATF2 (C-D) and cyclin D1 (G) in human GBM patient samples.
